# Metastatic skull base chordoma: A systematic review

**DOI:** 10.1002/lio2.906

**Published:** 2022-09-09

**Authors:** Kurtis Young, Torbjoern Nielsen, Hannah Bulosan, Tyler J. Thorne, Christian T. Ogasawara, Andrew C. Birkeland, Dennis M. Tang, Arthur W. Wu, Toby O. Steele

**Affiliations:** ^1^ University of Hawai'i at Mānoa John A. Burns School of Medicine Honolulu Hawaii USA; ^2^ Department of Neurosurgery University of Texas Medical Branch at Galveston Galveston Texas USA; ^3^ Department of Otolaryngology‐Head and Neck Surgery University of California Davis Medical Center Sacramento California USA; ^4^ Department of Otolaryngology‐Head and Neck Surgery Cedars‐Sinai Medical Center Los Angeles California USA

**Keywords:** chordoma, metastasis, rhinology, skull base, systematic review

## Abstract

**Objective/Hypothesis:**

To investigate the clinical features, management strategies and outcomes for patients with metastatic primary skull base chordomas.

**Study Design:**

Systematic review.

**Methods:**

A systematic search through Pubmed/Medline, Web of Science, and EBSCOhost (CINAHL) was conducted without restriction on dates. After study screening and full‐text assessment, two authors independently extracted all data using a pre‐established abstraction form.

**Results:**

Forty cases were included from 38 studies. The average age (standard deviation [SD]) of the sample at presentation was 28.5 (23.3) and was equally distributed across genders. The average time (SD) between initial diagnosis to local recurrence was 40.1 (60.3) months. The average time (SD) from primary tumor detection to the diagnosis of metastatic disease was 55.2 (49.0) months. The most common subsite for metastatic spread were the lungs (32.5%). Of the 33 patients with data on outcomes, 48.5% were found to have expired by the time of publication. The median overall survival was estimated to be 84 months (95% confidence interval [CI] 62.3–105.7).

**Conclusions:**

The most common subsites for metastatic spread of skull base chordoma were the lungs and bone. Overall survival for patients in the current cohort was a median of 84 months, with no significant differences noted when stratifying by the extent of surgery or the site of metastases.

**Level of Evidence:**

3a

## 1 INTRODUCTION

Chordomas are typically low‐grade neoplastic processes thought to arise from the notochord, making up 1–4% of all bone cancers.^1^, ^2^ Although chordomas were historically thought to arise most frequently in the sacrum, more recent literature has reported that the distribution is nearly equal between the sacrum (29.2%), mobile spine (32.8%), and skull base (32.0%).^3^ Chordomas have high rates of recurrence and are locally aggressive. Additionally, many of these skull base lesions are in close proximity to several vital neurovascular structures, including the brainstem, internal carotid arteries, cavernous sinuses, and several cranial nerves.^4^, ^5^ For these reasons, chordomas present a unique challenge to the skull base community.

The frequency with which chordomas metastasize has been estimated to be anywhere between 3% and 48%.^6^ However, these initial studies were conducted prior to 1980, and it is likely that the paucity of reported skull base lesions were a function of limitations in screening and technology.^7^, ^8^ There have been fewer investigations studying chordoma metastatic patterns in the 21st century, and these investigations have predominately examined neoplasms in the spine.^9^ Additionally, outcome data on local recurrences, distant metastases and survival of metastatic skull base (SB) chordomas are also limited. Furthermore, practical guidelines for the surgical and oncologic management of metastatic SB chordoma have yet to be defined. For instance, it is still unclear whether patients with distant SB chordoma metastases should be treated with primary resection, surgical removal of the metastasis, directed radiotherapy, or limited to palliative therapies. The present study is a systematic review designed to evaluate clinical outcomes of patients with SB chordoma that developed distant metastatic disease.

Following PICOS criteria (population, intervention, comparison, outcomes, study design), the current research question was developed: Regarding patients with SB chordoma with distant metastases, does the extent of surgery or site of metastasis influence survival outcomes? This investigation also had a secondary goal of synthesizing additional data on corresponding clinical features and management strategies of SB chordomas.

## 2 MATERIALS AND METHODS

### 2.1 Literature search and study screening

**FIGURE 1:**
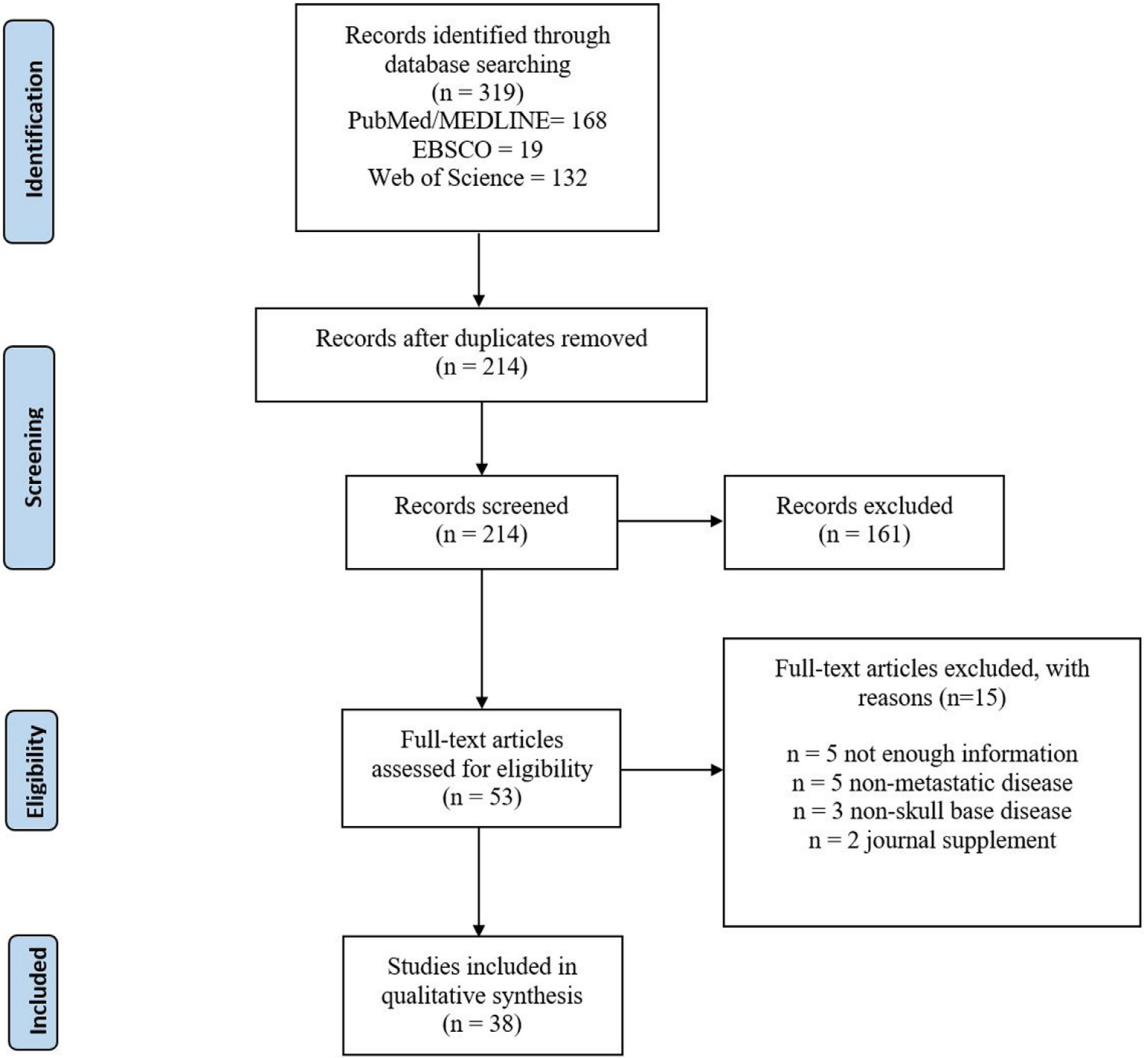
PRISMA flow diagram for search and review strategy

Author Kurtis Young conducted a systematic database search with no restrictions on years published across Pubmed/Medline, Web of Science, and CINAHL (EBSCOhost), following PRISMA guidelines. Several search phrases including, “skull base,” “chordoma” or “metastasis,” and several Boolean operators including “AND” or “OR” were combined in various permutations. This process and search parameters are fully described by Figure 1 and Appendix S1. The reference management software, Rayyan QCRI, was used to remove duplicates and to screen the initial group of studies. Authors Torbjoern Nielsen and Hannah Bulosan separately screened studies based on titles and abstracts through a blinded process. Author Kurtis Young reviewed and resolved all conflicts between the two aforementioned authors, and finalized all included studies through full‐text assessment. Inclusion criteria required that patients were diagnosed with primary skull base chordoma that subsequently metastasized either on presentation or throughout the course of the study. Additionally, included articles must have either been a case report, case series, or observational study with individual case data. Non‐English articles were excluded if they lacked an English language abstract or if that abstract did not have information on demographics, clinical presentation, and management/outcomes.

### 2.2 Data extraction

Authors Torbjoern Nielsen and Hannah Bulosan independently extracted data from the included articles through an abstraction form that was created beforehand, and authors Kurtis Young and Christian T. Ogasawara independently verified all extracted data. Data on study ID (publication year and author names), baseline demographics (age and gender), clinical features (presenting symptoms, primary site, recurrence data, and metastatic details), management (surgery, radiotherapy, and chemotherapy), and outcomes (overall survival, causes of death) were extracted. Meta‐analysis was not performed due to the level of evidence of the included studies (IV‐V), as determined following the 2011 Oxford Centre for Evidence‐Based Medicine guidelines.^10^

### 2.3 Data analysis

All analyses were performed utilizing SPSS software version 26 (IBM Corp., Armonk, New York). Means and standard deviations were calculated for continuous variables. The intervals between SB chordoma onset and death (OS curve) were approximated through Kaplan–Meier curves, with significance being assessed through the log‐rank test. Calculations were deemed to be statistically significant using an α of 0.05.

## 3 RESULTS

The systematic database search initially yielded 214 titles, with 53 studies that were further screened through full‐text assessment. Here, five and three studies were excluded for not featuring patients with metastatic disease or primary skull base tumors, respectively. Additionally, there were several studies (5) that were excluded for insufficient information. Two titles were found to be journal supplements and were excluded. Finally, there were 38 studies included in this review, with a total of 40 included cases.^11^, ^12^, ^13^, ^14^, ^15^, ^16^, ^17^, ^18^, ^19^, ^20^, ^21^, ^22^, ^23^, ^24^, ^25^, ^26^, ^27^, ^28^, ^29^, ^30^, ^31^, ^32^, ^33^, ^34^, ^35^, ^36^, ^37^, ^38^, ^39^, ^40^, ^41^, ^42^, ^43^, ^44^, ^45^, ^46^, ^47^, ^48^, ^49^ Study dates ranged from 1949–2022, with 10 studies being published prior to the 21st century. There were 9, 15, and 4 studies that were published between 2000–2009, 2010–2019, and 2020 onwards, respectively.

### 3.1 Quality appraisal

All included studies were case reports or case series and were subsequently classified as level V or IV evidence, respectively. The overall quality of the cases included were deemed to be moderate to high, as further detailed in Appendix S2. All studies reported on demographic data (100.0%), surgical management (100.0%), and local recurrences and metastases (100.0%). The majority of included cases featured information on symptoms associated with the primary tumor (77.5%), but many of the remaining patients were initially treated at different institutions and had unavailable prior medical history. Regarding outcomes, most patients (82.5%) presented with follow‐up data.

### 3.2 Demographic information and clinical characteristics

The average age (SD) on diagnosis of the primary tumor was 25.5 (21.7) years. The cohort was evenly distributed across genders. These data are more completely depicted in Table 1. Of the 31 patients with data on the symptoms at the time of primary tumor presentation, the most common reported findings were headaches (38.7%), neck pain or rigidity (32.3%), dysphagia or pharyngeal mass (29.0%), and vision impairment (22.6%). Less common symptoms included nausea (9.7%), vomiting (9.7%), seizure/epilepsy (6.5%), and gait impairment (6.5%). Cranial nerve (CN) impairment was noted in 12 of the 31 patients, with isolated impairment of CN VI (25%) or CN II (25%) being the most common. Several patients presented with either CN XII (16.7%) or combined CN VI and VII (16.7%) palsies. An additional patient presented with CN IX and CN XII impairment. Finally, one patient presented with palsies of CN IX, CN X, CN XI, and CN XII. All other symptoms fully listed in Table 1. The average duration (SD) between symptom presentation and diagnosis of the primary tumor was 11.1 (17.5) months. Finally, eight of 40 cases were reported as initially misdiagnosed. Further data on primary tumor management are represented in Appendix S3.

**TABLE 1:** Clinical data per the 40 cases with skull base chordoma

Author	Study year	Age (years)	Sex (M/F)	Presenting symptoms	Location primary	Surgery (primary)	Number of recurrences, treatment	Primary metastases, all metastases	Outcome, time from primary
Agrawal et al.	2008	32	M	Neck pain/rigidity	Petrous bone	Subtotal resection	3, P1: subtotal resection P2: subtotal resection P3: RT	Lungs, M1: lungs	Dead, NA
Agunbiade et al.	2020	9	M	Headache, nausea, vomiting, neck pain/rigidity, vision impairment, CN VI palsy, aniscoria, weight loss	Clivus	Unspecified resection	NA, NA	Lungs, M1: lungs	NA, NA
Asano et al.	2003	53	M	Vision impairment, dysphagia/pharyngeal mass	Clivus	Unspecified resection	2, P1: subtotal resection, GammaKnife P2: subtotal resection, RT	Bone, M1: T4, T8, T9, lumbar vertebra	Alive, 132
Auger et al.	1994	2	M	Dysphagia/pharyngeal mass, CN VI, VII palsy, slurred speech, dysphagia, irritability, bloody nasal discharge	Clivus	None	NA, NA	Lungs, M1: lungs	Dead, 0.75
Aydin et al.	2013	46	F	NA	Clivus	Unspecified resection	NA, NA	Bone, M1: T2, T3 M2: C1, C2	Alive, 150
Boyette et al.	2008	60	F	Headache	Clivus	Complete resection	3, P1: total resection, RT P2: total resection P3: NA	Soft tissue, M1: left retropharyngeal space	Dead, 69
Brooks et al.	1981	1.42	M	Dysphagia/pharyngeal mass, failure to thrive	Clivus	None	NA, NA	Lungs, M1: lungs	Dead, 7
Couldwell et al.	1996	8	F	Headache, nausea, vomiting, neck pain/rigidity, gait impairment, dysphagia/pharyngeal mass	Clivus, occipital condyle, left posterior fossa	Subtotal resection	2, P1: total resection P2: total resection	Soft tissue, M1: subcutaneous nodules in back, Abdomen	Alive, 28
Dahl et al.	2017	0.17	M	Seizure/epilepsy, developmental delay, decreased oral intake, macrocephaly	Clivus	Biopsy only	NA, NA	Soft tissue, M1: chest wall	NA, NA
Figueiredo et al.	2011	18	M	NA	“Pineal mass”	Unspecified resection	1, none	Soft tissue, M1: abdomen (mesogastrium)	Dead, NA
Fischbein et al.	2000	47	M	Vision impairment	Clivus	Complete resection	1, GammaKnife	Soft tissue, M1: subcutaneous mass right bridge of nose	Alive, 77
Fischbein et al.	2000	47	M	NA	Clivus	Subtotal resection	0, NA	Soft tissue, M1: anterior nasal mass	Alive, 60
Fischbein et al.	2000	33	F	Headache, vision impairment	Clivus	Subtotal resection	2, P1: subtotal resection P2: resection, RT	Bone, M1: anterior ethmoid	Alive, 84
Goes et al.	2015	52	Not stated	NA	Clivus	Subtotal resection	1, P1: resection, RT	Soft tissue, M1: subcutaneous medial right neck M2: C4, C5	Alive, 48
Hines et al.	2014	35	F	NA	Clivus	Subtotal resection	1, P1: nonoperative, medical management	Soft tissue, M1: left nasal cavity	NA, NA
Iloreta et al.	2014	47	M	Upper back pain	Clivus to C5	Complete resection	1, P1: Resection, RT	Soft tissue, M1: anterior left neck incision site	NA, NA
Jain et al.	2013	30	F	Headache, dysphagia/pharyngeal mass, dysarthria, sore throat	Skull base near foramen magnum to C1, C2	None	NA, NA	Soft tissue, M1: nasopharynx	NA, NA
Kaneko et al.	1991	4	F	Neck pain/rigidity, vision impairment, gait impairment, CN VI, VII palsy	Clivus	Subtotal resection	1, P1: resection	Disseminated, M1: dura mater M2: skull bone M3: lungs M4: liver M5: sternum M6: left humerus M7: vertebrae	dead, 9
Kearns et al.	2016	3	M	Neck pain/rigidity	Edge of foramen magnum, clivus	Complete resection	2, P1: total resection P2: chemotherapy (Imatinib followed by etoposide)	Lungs, M1: lungs M2: subcutaneous tissue, neck at incision site	Dead, 48
Kim et al.	2018	32	F	Dysphagia/pharyngeal mass	Clivus, right jugular tubercule, retropharyngeal space	Complete resection	0, NA	Soft tissue, M1: heart	Dead, NA
Krishnamurthy et al.	2020	7	M	Left hemiface swelling	Sella, clivus	Unspecified resection	NA, NA	Bone, M1: left hemimandible M2: lungs M3: right mandible	Dead, 4
Loehn et al.	2009	33	F	Headache, vision impairment	Spheno‐occipital	Unspecified resection	1, P1: chemotherapy, RT, resection	Soft tissue, M1: neck M2: mandible M3: lungs M4: vertebral column M5: axial skeleton	Dead, 84
Lopez et al.	2022	29	M	Headache	Clivus	Subtotal resection	NA, NA	Bone, M1: thoracic and lumbar spine M2: distal spinal cord and cauda equina nerve roots	Alive, 26
Lountzis et al.	2006	1.67	Not stated	NA	Clivus	None	NA, NA	Disseminated, M1: skin M2: spinal cord M3: lungs M4: heart M5: liver M6: kidneys M7: brain parenchyma	Alive, 30
Maira et al.	1996	80	F	Vision impairment, CN VI palsy, pituitary endocrinopathy	Clivus, sphenoid sinus, sella (multiple primaries)	Complete resection	NA, NA	Bone, M1: lumbar spine	Alive, 18
Martin et al.	2009	69	M	NA	Clivus	Unspecified resection	1, NA	Bone, M1: L4, L5, cauda equina	Alive, 144
Nor et al.	2018	59	F	Vision impairment, CN II palsy	Seller, supraseller	Complete resection	NA, NA	Bone, M1: foramen magnum M2: posterior epidural space L4, L5	Alive, 11
Ogi et al.	1995	24	F	Headache, dysphagia/pharyngeal mass, CN IX, X, XI, XII palsy	Clivus	Subtotal resection	1, NA	Soft tissue, M1: skin M2: lungs, lymph nodes M3: pelvic cavity	Dead, 42
Plese et al.	1978	6	F	Seizure/epilepsy, CN II palsy	Clivus	Subtotal resection	NA, NA	Soft tissue, M1: subarachnoid space brain and spinal cord	Dead, 0
Renard et al.	2014	2	F	Neck pain/rigidity, CN VI palsy	Clivus	Biopsy only	NA, NA	Bone, M1: right costovertebral gutter	Alive, 27
Rutkowski et al.	2017	5	M	Headache, neck pain/rigidity, dysphagia/pharyngeal mass, CN XII palsy, dyspnea, decline in appetite and oral intake, decreased gustatory sensation, speech hesitation, dysarthria	Clivus	Unspecified resection	NA, NA	Lungs, M1: lungs M2: chest wall M3: calvaria M4: skin M5: chest wall	Alive, 70
Schonegger et al.	2005	51	M	Difficulty breathing through nose	Clivus infiltrating sinus sphenoid	Complete resection	3, P1: resection P2: resection P3: resection, RT, etoposide, ifosfamide, doxorubicin, vincristine, isotretinoin, interferon‐alpha	Lungs, M1: lungs M2: brain	Alive, 108
Shakir et al.	2016	68	F	Headache, dysphagia/pharyngeal mass, CN XII palsy, imbalance, tongue weakness, imbalance	Foramen magnum	Subtotal resection	NA, NA	Soft tissue, M1: breast	NA, NA
Sibley et al.	1987	2	F	Neck pain/rigidity, fever, irritability, anorexia	Spheno‐occipital ‐vertebral	None	NA, NA	Lungs, M1: lungs	Dead, 3
Uggowitzer et al.	1999	22	M	CN IX, XII palsy, hypoacusis, vertigo	Clivus	Subtotal resection	1, NA	Bone, M1: C4/5 intervertebral foramen M2: C5/6 intervertebral foramen M3: thoracic and lumbar spine	Alive, 78
Uhr et al.	1949	21	M	Headache, nausea, vomiting, neck pain/rigidity, constipation, anorexia, diminished hearing, weight loss, weakness, fever, limb pain	Sella turcica	None	NA, NA	Lungs, M1: lungs	Dead, 6
van Lierop et al.	2008	18	F	NA	Clivus	Subtotal resection	NA, NA	Bone, M1: hard palate	Alive, 24
Yasue et al.	2022	2	F	Neck pain/rigidity, unable to move left upper limb, dyspnea	Clivus	Biopsy only	1, P1: resection, RT	Bone, M1: left upper arm M2: right iliac bone	Dead, NA
Zemmoura et al.	2012	42	M	Headache, vision impairment, CN II palsy	Clivus	Subtotal resection	2, P1: resection P2: resection	Bone, M1: maxilla	Dead, 84
Zener et al.	2011	59	F	Neck pain/rigidity	Clivus	Unspecified resection	NA, NA	Soft tissue, M1: left mid‐sternocleidomastoid region	NA, NA

### 3.3 Metastatic and locally recurrent disease management

All of the cases included in this study developed metastatic disease, and 37.5% of the sample experienced distant spread to more than one metastatic site. The lungs were the most common subsite involved overall (32.5%) and were the most common subsite for isolated metastatic spread, accounting for 15% of the sample. There were 30 cases of bony metastases, with the most common sites being the lumbar (20.0%), thoracic (13.3%), and cervical spine (10%). The average time (SD) from primary tumor detection to the diagnosis of metastatic disease was 55.2 (49.0) months. Subsequently, the average time (SD) from the detection of first distant metastatic disease to additional distant metastases was 16.6 (13.8) months. The most common imaging modalities used in confirming these metastases were CT and MRI, as more completely depicted by Appendix S4. There were 29 cases where biopsy of the metastatic lesion was performed, confirming the diagnosis of metastatic SB chordoma. Treatment of metastatic disease was dependent on the location of the malignant spread, as detailed in Appendix S4.

Of the primary lesions that were initially surgically treated, 20 were found to have recurred at the primary site. While the majority of these patients reported a single recurrence (55.0%), there were several that presented with two (25.0%) or three (15.0%) separate recurrences. Of the 13 patients with data on the treatment of locally recurrent SB chordoma, the most common treatment modality was re‐excision (84.6%), and other management strategies (Appendix S5). The average time (SD) between the initial diagnosis to the first local recurrence was 40.1 (60.3) months. The average time (SD) between the first recurrence and second local recurrence was found to be 24.3 (6.1) months.

### 3.4 Survival outcomes

**FIGURE 2:**
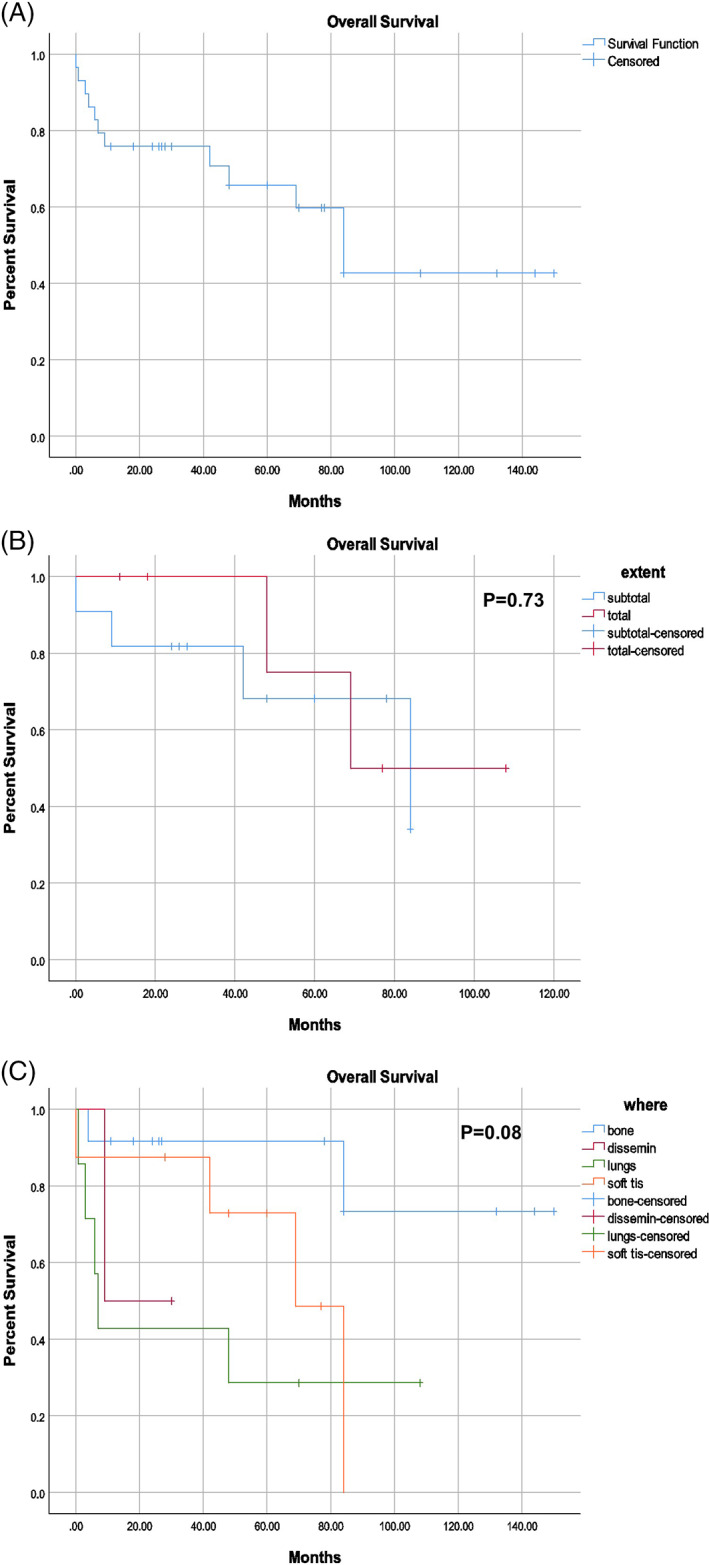
Kaplan–Meier curves depicting overall survival in the (A) full cohort, (B) groups separated by the extent of surgery, and (C) groups separated by the site of primary metastasis

There were data on survival outcomes for 33 patients (82.5%), 17 of which were alive (51.5%) on follow‐up and 16 (48.5%) of which were reported to have expired. There were seven cases (17.5%) where no survival data were available. The causes of death were variable and are more clearly shown in Table 2. The average follow‐up time (SD) for patients reported as being alive was 65.6 (45.5) months after primary detection. The median overall survival since the diagnosis of the primary lesion was estimated to be 84 months (95% CI 62.3–105.7). The median overall survival lowers to 48 months (95% CI 23.7–72.3) from the diagnosis of metastatic disease. Differences in overall survival based on the extent of surgery (total versus sub‐total) were not of statistical significance. Additionally, there were no statistically significant differences noted regarding overall survival across different metastatic sites. These survival data are better illustrated by the Kaplan–Meier curves featured in Figure 2.

**TABLE 2:** Outcomes of metastatic skull base chordomas

Authors	Survival from primary detection (months)	Survival from metastasis detection (months)	Status	Cause of death?
Fischbein et al.	60	36	Alive	NA
Fischbein et al.	77	28	Alive	NA
Goes et al.	48	12	Alive	NA
Martin et al.	144	0	Alive	NA
Uggowitzer et al.	78	36	Alive	NA
Asano et al.	132	0	Alive	NA
Couldwell et al.	28	0	Alive	NA
Fischbein et al.	84	84	Alive	NA
Schonegger et al.	108	24	Alive	NA
Aydin et al.	150	90	Alive	NA
Lopez et al.	26	2	Alive	NA
Lountzis et al.	30	30	Alive	NA
Maira et al.	18	ns	Alive	NA
Nor et al.	11	0	Alive	NA
Renard et al.	27	9	Alive	NA
Rutkowski et al.	70	15	Alive	NA
van Lierop et al.	24	0	Alive	NA
Kim et al.	NA	NA	Dead	Tumor progression and heart failure
Figueiredo et al.	NA	NA	Dead	NA
Kaneko et al.	9	0	Dead	NA
Loehn et al.	84	36	Dead	NA
Ogi et al.	42	30	Dead	NA
Yasue et al.	NA	NA	Dead	Meningitis
Kearns et al.	48	48	Dead	Brainstem herniation
Zemmoura et al.	84	53	Dead	Infection
Agrawal et al.	ns	ns	Dead	Aspiration pneumonia
Boyette et al.	69	28	Dead	Recurrence
Auger et al.	0.75	0.75	Dead	Massive pulmonary embolism
Brooks et al.	7	ns	Dead	NA
Krishnamurthy et al.	4	4	Dead	NA
Plese et al.	0	0	Dead	Hypovolemic shock
Sibley et al.	3	3	Dead	NA
Uhr et al.	6	6	Dead	Respiratory failure

## 4 DISCUSSION

Although the primary objective of this systematic review was to examine the clinical features, management, and outcomes of metastatic SB chordoma, there are several key points to be made regarding the workup and treatment of the primary tumor. Diagnosing SB chordomas may be challenging secondary to the plethora of nonspecific symptoms on presentation, and diagnosis is often rendered at later stages after the tumor has already compressed or invaded adjacent structures. Indeed, even the most common symptoms including headache, neck pain or rigidity, and dysphagia were noted in <50% of cases and were nonspecific to SB chordoma. The insidious, slow‐growing nature of SB chordomas may be the reason why many patients do not initially present symptomatically, explaining why even the most common symptoms are detected relatively infrequently. Many patients with SB chordoma in the current study were initially misdiagnosed (20.0%), leading to longer intervals before diagnosis. In the current study, the time from initial symptom onset to diagnosis was an average of 11.1 months. While chordomas are slowly growing malignancies, earlier management is integral in promoting favorable patient prognoses and outcomes.^50^

The main principles in primary SB chordoma management are local control and the prevention of distant metastatic spread.^51^ Therefore, total macroscopic resection should be performed to reduce the chance for disease recurrence or metastases.^52^ While only 31 cases received surgical management for their primary tumor, nearly 65% of these patients experienced local recurrence. In contrast, the literature state that the 5‐year recurrence rates ranged anywhere between 19% and 54%, and this was independent of surgical margin status or adjuvant radiotherapy.^51^ This difference may be explained by the included metastasis‐bound SB chordomas potentially having more aggressive phenotypes when compared with a more general SB chordoma sample. However, the information on tumor size, histologic and mutational features were far too limited to confirm this hypothesis. There have been limited literature regarding prognosticators in chordoma recurrence, but Pallini et al. found that expression of human telomerase reverse transcriptase (hTERT) was reliable in estimating tumor aggression. Additionally, the expression of brachyury and the loss of the SWI/SNF chromatin remodeling factor subunit (INI1), has been associated with poorly differentiated chordomas, which are also associated with poorer outcomes. In the current review, first‐time recurrences were most often re‐excised (84.6%). However, due to the rarity of SB chordomas, the data on treatment is scarce and no firm guidelines have been established on how to manage these chemoradiotherapy‐resistant recurrent lesions.

While chordomas have long been characterized as being radiotherapy resistant, survival benefits have been reported across several studies.^52^, ^53^, ^54^ Although chordomas have been traditionally unresponsive to cytotoxic chemotherapy, newer targeted immunotherapeutic options, have shown promising results.^55^ For instance, in patients with PDGFRβ‐positive chordomas, the first and second‐line therapies are tyrosine‐kinase (imatinib) and epidermal growth factor receptor (EGFR) inhibitors (erlotinib), respectively.^56^, ^57^ Additionally, the combination of cetuximab and gefitinib, both EGFR inhibitors, have demonstrated to be effective in the treatment of chordomas.^58^, ^59^ There have been modest data supporting the efficacy of lapatinib, a dual inhibitor for EGFR and erbB‐2/human epidermal growth factor receptor 2 (HER2/neu), in patients positive for HER2/neu expression.^60^ Bevacizumab, a vascular endothelial growth factor (VEGF) inhibitor, has demonstrated promising results when used in conjunction with erlotinib therapy, as VEGF levels have been found to be substantially in chordoma tissues.^61^, ^62^ The prognosis for poorly differentiated chordomas (PDC), characterized by the expression of brachyury and loss INI1, is significantly worse compared with their conventional counterparts, and treatment options for this condition are sparse.^63^ While there has been a recent clinical trial has investigated the role of Tazemetostat in treating adolescents and adults with PDC, the majority of patients with this condition are typically pediatric.^64^ Although there have been other trials investigating other target treatment molecules including nivolumab, these studies were limited by small samples.^63^ Additional research on molecular markers, genomic aberrancies, and molecular targeted therapies are needed in the treatment of PDCs. However, the ability to conduct trials on prognostic biomarkers and new therapeutic agents may be challenging due to the rarity of this disease.

This systematic review marks the first attempt to gather and collate cases of metastatic primary SB chordoma, an entity that has been very rarely reported in the literature. SB chordomas are rare malignancies that often present with nonspecific and infrequent symptomatic patterns. Unfortunately, many patients were found to face significant delays in diagnosis, which may have contributed to the eventual development of metastatic disease. According to the present study, the most common subsite for metastatic spread were the lungs, which is concordant with the literature.^65^ Similarly, many metastases were found along several segments of the spine, including the lumbar (6), thoracic (4), cervical (3), and other, unspecified vertebrae (4). In the current systematic review, the average time from diagnosis to the detection of distant metastases was found to be 55.2 months, which was similar to the median of 58.3 months reported in a retrospective study of metastatic chordomas by Young et al.^65^ The present study found that the primary site of metastasis with the best prognosis to be the bone, although this result was outside of statistical significance (*p* = .08). On the contrary, Young et al. found the primary site with the worst prognosis to be the bone, with soft tissue and lung primary metastatic sites to have better survival outcomes. However, it should be noted that the sample (*n* = 219) these researchers used were predominately based in the sacrum (60.7%) and mobile spine (34.7%), with few primary tumors of the skull base (1.8%).^65^ This variability may explain the discrepancies in distant metastatic sites between the current study and the investigation lead by these researchers. Larger, prospective trials are necessary to delineate the differences between the patterns of metastatic spread and survival outcomes between the skull base, mobile spine, and sacrum.

The prognosis for a general population of SB chordomas without predetermined metastatic involvement is generally better than that of this study. Unfortunately, most of the studies in the literature are limited by small sample sizes, secondary to the rare nature of this disease. In comparison to the findings of the current study,^65^ the 5, 10, and 15‐year OS for a more general sample of 24 patients with SB chordomas were found to be 86%, 72%, and 72%.^66^ In separate sample of 24 patients with clival chordomas, the 5‐ and 10‐year OS were found to be 69% and 60%, respectively.^52^ In Chambers et al.'s study of 594 cases derived from the Surveillance, Epidemiology, and End Results (SEER) program, the median overall survival of patients with cranial chordomas was found to be 9.2 years. It is important to note that this study found that the 5‐year OS for these patients significantly increased from different eras, with values of 48.5%, 73.5%, and 80.7% from 1975 to 1984, 1985 to 1994, and 1995 to 2004, respectively (*p* < .01).^67^ In contrast, the current study estimated that the median OS for this cohort with primary SB chordomas that eventually developed metastatic disease to be lower at 7 years. Additionally, it is important to note that the current study also included cases up until 2022, which may lead to improved survival outcomes when compared with the investigation by Chambers et al. Nonetheless, further analysis and comparison with the current sample is precluded by available data on an uncommon subset of a rare condition. Further research is needed to determine the differences between the features of metastatic chordomas that lead to more aggressive behavior than when compared with more conventional types. More comprehensive data on properties including histologic grade, histologic subtype, tumor size, and molecular features are needed.^68^

This systematic review had several limitations. First, the data were limited to case reports and small series, with nonsystematic reporting of the data. Additionally, since all cases featured SB chordomas that eventually metastasized, some data (including those on recurrences or the extent of surgery) may be inherently skewed. Furthermore, finer granularity data on the specific areas of clival involvement were not available in most of the included investigations, preventing additional analyses. Finally, the data pertaining to specific surgical approaches and adjuvant therapies were highly variable, precluding further subgroup analysis through the Kaplan–Meier method. However, this study is not without any strengths. These included a multiple author systematic database search strategy, study screening, and data extraction and synthesis. Additionally, this study identifies a specific area within the skull base field in need of further research. Most importantly, this is the first systematic review to investigate a rare subset of exceedingly aggressive skull base malignancies. Further multi‐institutional prospective research is needed to fully elucidate the clinical patterns and outcomes of this challenging skull base malignancy.

## 5 CONCLUSION

Pulmonary and spinal metastasis were the most common sites of metastatic spread of SB chordoma. Overall survival was a median of 84 months, but no significant differences in survival outcomes were noted when stratifying the sample by the extent of surgery or the site of metastases. Overall survival was found to be lower in the current metastatic subgroup compared with more general SB chordoma populations.

## CONFLICT OF INTEREST

The authors declare no conflict of interest.

## Supporting information


**APPENDIX S1** Search methodology

**APPENDIX S2** Included cases with quality appraisal^1^, ^2^, ^3^, ^4^, ^5^, ^6^, ^7^, ^8^, ^9^, ^10^, ^11^, ^12^, ^13^, ^14^, ^15^, ^16^, ^17^, ^18^, ^19^, ^20^, ^21^, ^22^, ^23^, ^24^, ^25^, ^26^, ^27^, ^28^, ^29^, ^30^, ^31^, ^32^, ^33^, ^34^, ^35^, ^36^, ^37^, ^38^

**APPENDIX S3** Surgical management of primary skull base chordoma

**APPENDIX S4** Further information on skull base chordoma metastases

**APPENDIX S5** Further information on skull base chordoma recurrences
