# Supplementary material for: Metastatic skull base chordoma: A systematic review
Source: Laryngoscope Investig Otolaryngol. 2022 Sep 9;7(5):1280–91. doi: 10.1002/lio2.906 (PMC9575061; doi:10.1002/lio2.906)
Supplement: Supplementary file 1 — APPENDIX S1 Search methodology [file LIO2-7-1280-s003.docx]

**Appendix S1: Search Methodology**

Pubmed: ("skull base"[All Fields] OR "skull"[All Fields] OR "cranial base"[All Fields] OR "clivus"[All Fields] OR "clival"[All Fields]) AND "chordoma"[All Fields] AND ("metastasis"[All Fields] OR "metastases"[All Fields] OR "metastatic"[All Fields] OR "surgical pathway"[All Fields]) AND ("case"[All Fields] OR "cases"[All Fields])

Web of Science: ("skull base" OR "skull" OR "cranial base" OR "clivus" OR "clival") AND ("chordoma") AND ("metastasis" OR "metastases" OR "metastatic" OR "surgical pathway") AND ("case" OR "cases")

EBSCOhost (CINAHL): ("skull base" OR "skull" OR "cranial base" OR "clivus" OR "clival") AND ("chordoma") AND ("metastasis" OR "metastases" OR "metastatic" OR "surgical pathway") AND ("case" OR "cases")
