# Supplementary material for: Metastatic skull base chordoma: A systematic review
Source: Laryngoscope Investig Otolaryngol. 2022 Sep 9;7(5):1280–91. doi: 10.1002/lio2.906 (PMC9575061; doi:10.1002/lio2.906)
Supplement: Supplementary file 2 — APPENDIX S2 Included cases with quality appraisal 1 , 2 , 3 , 4 , 5 , 6 , 7 , 8 , 9 , 10 , 11 , 12 , 13 , 14 , 15 , 16 , 17 , 18 , 19 , 20 , 21 , 22 , 23 , 24 , 25 , 26 , 27 , 28 , 29 , 30 , 31 , 32 , 33 , 34 , 35 , 36 , 37 , 38 [file LIO2-7-1280-s005.docx]

| **Appendix S2. Included Cases with Quality Appraisal^1-38^** | | | | | | | | | | |
| --- | --- | --- | --- | --- | --- | --- | --- | --- | --- | --- |
| Ref | Year Published | Level of Evidence | Age (years) | Sex (M/F) | Presenting symptoms | Surgery (primary) | Recurrences | Metastases | Imaging of Metastases | Outcomes |
| 1 | 2008 | V | 32 | m | yes | yes | yes | yes | yes | yes |
| 2 | 2020 | V | 9 | m | yes | yes | none | yes | yes | not stated |
| 3 | 2003 | V | 53 | m | yes | yes | yes | yes | yes | yes |
| 4 | 1994 | V | 2 | m | yes | no | none | yes | yes | yes |
| 5 | 2013 | V | 46 | f | not stated | yes | none | yes | yes | yes |
| 6 | 2008 | V | 60 | f | yes | yes | yes | yes | yes | yes |
| 7 | 1981 | V | 1.42 | m | yes | no | none | yes | no | yes |
| 8 | 1996 | V | 8 | f | yes | yes | yes | yes | yes | yes |
| 9 | 2017 | V | 0.17 | m | yes | no | none | yes | not stated | not stated |
| 10 | 2011 | V | 18 | m | not stated | yes | yes | yes | yes | yes |
| 11 | 2000 | IV | 47 | m | not stated | yes | yes | yes | yes | yes |
| 11 | 2000 | IV | 47 | m | yes | yes | none | yes | yes | yes |
| 11 | 2000 | IV | 33 | f | yes | yes | yes | yes | yes | yes |
| 12 | 2015 | V | 52 | not stated | yes | yes | yes | yes | yes | yes |
| 13 | 2014 | V | 35 | f | not stated | yes | yes | yes | yes | not stated |
| 14 | 2014 | V | not stated | m | not stated | yes | yes | yes | yes | not stated |
| 15 | 2013 | V | 30 | f | yes | no | none | yes | yes | not stated |
| 16 | 1991 | V | 4 | f | yes | yes | yes | yes | no | yes |
| 17 | 2016 | V | 3 | m | yes | yes | yes | yes | yes | yes |
| 18 | 2018 | V | 32 | f | yes | yes | none | yes | yes | yes |
| 19 | 2020 | V | 7 | m | yes | yes | none | yes | yes | yes |
| 20 | 2009 | V | 33 | f | yes | yes | yes | yes | yes | yes |
| 21 | 2022 | V | 29 | m | yes | yes | none | yes | yes | yes |
| 22 | 2006 | V | 1 | not stated | not stated | no | none | yes | not stated | yes |
| 23 | 1996 | V | 80 | f | yes | yes | none | yes | not stated | yes |
| 24 | 2009 | V | 69 | m | not stated | yes | yes | yes | yes | yes |
| 25 | 2018 | V | 59 | f | yes | yes | none | yes | yes | yes |
| 26 | 1995 | V | 24 | f | yes | yes | yes | yes | yes | yes |
| 27 | 1978 | V | 6 | f | yes | yes | none | yes | no | yes |
| 28 | 2014 | V | 2 | f | yes | no | none | yes | not stated | yes |
| 29 | 2017 | V | 5 | m | yes | yes | none | yes | yes | yes |
| 30 | 2005 | V | 51 | m | yes | yes | yes | yes | yes | yes |
| 31 | 2016 | V | 68 | f | yes | yes | none | yes | yes | not stated |
| 32 | 2020 | V | 5 | f | not stated | yes | none | yes | not stated | yes |
| 33 | 1999 | V | 22 | m | yes | yes | none | yes | yes | yes |
| 34 | 1949 | V | 21 | m | yes | no | yes | yes | yes | yes |
| 35 | 2008 | V | 18 | f | not stated | yes | none | yes | not stated | yes |
| 36 | 2022 | V | 2 | f | yes | no | none | yes | yes | yes |
| 37 | 2012 | V | 42 | m | yes | yes | yes | yes | yes | yes |
| 38 | 2011 | V | 59 | f | not stated | yes | yes | yes | yes | not stated |

References

1. A A, Agrawal A. Chondroid chordoma of petrous temporal bone with extensive recurrence and pulmonary metastases. *Journal of Cancer Research & Therapeutics*. 2008-04- 2008;4(2):91-92.

2. Agunbiade S, Nada A, Bhimaniya S, Whitehead MT, Mahdi ES. Chordoma with lung metastases at initial presentation of a pediatric patient. *Radiol Case Rep*. 2020 2020;15(4):382-386.

3. Asano S, Kawahara N, Kirino T, Asano S, Kawahara N, Kirino T. Intradural spinal seeding of a clival chordoma. *ACTA NEUROCHIRURGICA*. 2003-7 2003;145(7):599-603.

4. Auger M, Raney B, Callender D, Eifel P, Ordóñez NG. Metastatic intracranial chordoma in a child with massive pulmonary tumor emboli. *Pediatr Pathol*. 1994 1994;14(5):763-70.

5. Aydin AL, Sasani M, Oktenoglu T, Solaroglu I, Ozer AF. A Case of Chordoma Invading Multiple Neuroaxial Bones: Report of Ten Years Follow Up. *Turkish Neurosurgery*. 2013 2013;23(4):551-556.

6. Boyette J, Seibert J, Fan C, et al. The etiology of recurrent chordoma presenting as a neck mass: Metastasis vs. surgical pathway seeding. *ENT-EAR NOSE & THROAT JOURNAL*. 2008-2 2008;87(2):106-109.

7. LJ B, E A, C H, J F. *Clivus chorodoma with pulmonary metastases appearing as failure to thrive*. Vol. 135. 1981:713-5. 0002-922X (Print). 1981-8. <https://pubmed.ncbi.nlm.nih.gov/7270512/>

8. Couldwell W, Stillerman C, Rice D, et al. Malignant clival chordoma with postoperative cutaneous metastases. *SKULL BASE SURGERY*. 1996-1 1996;6(1):61-66.

9. NA D, T L, M L, et al. *Chordoma Occurs in Young Children With Tuberous Sclerosis*. Vol. 76. 2017:418-423. 1554-6578 (Electronic). 2017-6-1. <https://pubmed.ncbi.nlm.nih.gov/28498973/>

10. Figueiredo EG, Tavares WM, Welling L, Rosemberg S, Teixeira MJ. Ectopic pineal chordoma. *Surg Neurol Int*. 2011 2011;2:145.

11. NJ F, MJ K, RA H, WP D. *Recurrence of clival chordoma along the surgical pathway*. Vol. 21. 2000:578-83. 0195-6108 (Print). 2000-3. <https://pubmed.ncbi.nlm.nih.gov/10730655/>

12. Goes R, van Overbeeke JJ. A vertebral extra dural chordoma at C5, possibly deriving from a clival chordoma. *Surg Neurol Int*. 2015 2015;6:94.

13. Hines J, Ashmead M, Stringer S, Hines JP, Ashmead MG, Stringer SP. Clival chordoma of the nasal septum secondary to surgical pathway seeding. *AMERICAN JOURNAL OF OTOLARYNGOLOGY*. 2014 2014;35(3):431-434.

14. Iloreta AM, Nyquist GG, Friedel M, Farrell C, Rosen MR, Evans JJ. Surgical pathway seeding of clivo-cervical chordomas. *J Neurol Surg Rep*. 2014 2014;75(2):e246-50.

15. Jain B, Datta S, Roy S, et al. Skull base chordoma presenting as nasopharyngeal mass with lymph node metastasis. *JOURNAL OF CYTOLOGY*. 2013 2013;30(2):145-147.

16. KANEKO Y, SATO Y, IWAKI T, et al. CHORDOMA IN EARLY-CHILDHOOD - A CLINICOPATHOLOGICAL STUDY. *NEUROSURGERY*. 1991-9 1991;29(3):442-446.

17. C K, C K. *Fifty-four-month survival in a 3-year-old child presenting with an aggressive metastatic dedifferentiated clival chordoma*. Vol. 2016. 2016. 1757-790X (Electronic). 2016-6-9. <https://pubmed.ncbi.nlm.nih.gov/27284102/>

18. Kim JW, Hong CK, Cha YJ, Kim SH, Suh CO, Lee KS. Cardiac Metastasis from Clivus Chordoma. *World Neurosurgery*. 2018 2018;113:103-107.

19. Krishnamurthy A. Mandibular metastasis as a presenting feature of a clival chordoma. 2020-04- 2020;16(2):668-671.

20. Loehn B, Walvekar R, Harton A, et al. Mandibular Metastasis from a Skull Base Chordoma: Report of a Case with Review of Literature. *SKULL BASE-AN INTERDISCIPLINARY APPROACH*. 2009-9 2009;19(5):363-368.

21. Lopez O, Al Ashi A, Izquierdo-Pretel G. Metastatic Clivus Chordoma: A Case of a Rare Tumor in a 29-Year-Old African American Male. *Cureus Journal of Medical Science*. 2022 2022;14(1)

22. Lountzis NI, Hogarty MD, Kim HJ, Junkins-Hopkins JM. Cutaneous metastatic chordoma with concomitant tuberous sclerosis. *Journal of the American Academy of Dermatology*. 2006 2006;55(2):S6-S10.

23. Maira G, Pallini R, Anile C, et al. Surgical treatment of clival chordomas: The transsphenoidal approach revisited. *JOURNAL OF NEUROSURGERY*. 1996-11 1996;85(5):784-792.

24. MP M, S O. *Intradural drop metastasis of a clival chordoma*. Vol. 16. 2009:1105-7. 0967-5868 (Print). 2009-8. <https://pubmed.ncbi.nlm.nih.gov/19410463/>

25. Nor F, Desai V, Chew L, Nor FEM, Desai V, Chew LL. Clival chordoma with drop metastases. *JOURNAL OF RADIOLOGY CASE REPORTS*. 2018-3 2018;12(3):1-9.

26. Ogi H, Kiryu H, Hori Y, et al. Cutaneous metastasis of CNS chordoma. *AMERICAN JOURNAL OF DERMATOPATHOLOGY*. 1995-12 1995;17(6):599-602.

27. Plese JP, Borges JM, Nudelman M, Lefèvre AB, Sallum J. Unusual subarachnoid metastasis of an intracranial chordoma in infancy. *Childs Brain*. 1978 1978;4(4):251-6.

28. Renard C, Pissaloux D, Decouvelaere AV, Bourdeaut F, Ranchere D. Non-rhabdoid pediatric SMARCB1-deficient tumors: overlap between chordomas and malignant rhabdoid tumors? *Cancer Genetics*. 2014 2014;207(9):384-389.

29. Rutkowski M, Birk H, Wood M, et al. Metastatic clival chordoma: a case report of multiple extraneural metastases following resection and proton beam radiotherapy in a 5-year old boy. *JOURNAL OF NEUROSURGERY-PEDIATRICS*. 2017-5 2017;19(5):531-537.

30. K S, E G, D P, et al. *Recurrent and metastatic clivus chordoma: systemic palliative therapy retards disease progression*. Vol. 16. 2005:1139-43. 0959-4973 (Print). 2005-11. <https://pubmed.ncbi.nlm.nih.gov/16222158/>

31. Shakir S, Pelmus M, Florea A, et al. Synchronous metastatic skull base chordoma to the breast: case report and literature review. *CURRENT ONCOLOGY*. 2016-4 2016;23(2):E154-E157.

32. Sibley RK, Day DL, Dehner LP, Trueworthy RC. Metastasizing chordoma in early childhood: a pathological and immunohistochemical study with review of the literature. *Pediatr Pathol*. 1987 1987;7(3):287-301.

33. Uggowitzer M, Kugler C, Groell R, et al. Drop metastases in a patient with a chondroid chordoma of the clivus. *NEURORADIOLOGY*. 1999-7 1999;41(7):504-507.

34. N U, J C. Hypertrophic osteoarthropathy; report of a case associated with a chordoma of the base of the skull and lymphangitic pulmonary metastases. *Annals of internal medicine*. 1949-10 1949;31(4):681-91.

35. van Lierop AC, Fagan JJ, Taylor KL. Recurrent chordoma of the palate occurring in the surgical pathway: A case report. *Auris Nasus Larynx*. 2008 2008;35(3):447-450.

36. Yasue S, Ozeki M, Endo S, et al. Poorly Differentiated Chordoma of the Clivus With Loss of SMARCB1 Expression in a Pediatric Patient: A Case Report. *J Pediatr Hematol Oncol*. 2022 2022;

37. Zemmoura I, Ben Ismail M, Travers N, Jan M, François P. Maxillary surgical seeding of a clival chordoma. *Br J Neurosurg*. 2012 2012;26(1):102-3.

38. Zener R, Jacquet Y, Wong JW, Enepekides D, Higgins KM. A rare case of surgical pathway implantation of clival chordoma presenting as a neck mass. *J Surg Case Rep*. 2011 2011;2011(1):3.
