# Supplementary material for: Metastatic skull base chordoma: A systematic review
Source: Laryngoscope Investig Otolaryngol. 2022 Sep 9;7(5):1280–91. doi: 10.1002/lio2.906 (PMC9575061; doi:10.1002/lio2.906)
Supplement: Supplementary file 3 — APPENDIX S3 Surgical management of primary skull base chordoma [file LIO2-7-1280-s001.docx]

| **Appendix S3.** Surgical Management of Primary Skull Base Chordoma | | | | | | |
| --- | --- | --- | --- | --- | --- | --- |
| Authors | Location Primary | Surgery Extent | Surgical Approach | Surgical Complication | Histology Type | Molecular features |
| Agrawal et al. | petrous bone | subtotal resection | NA | CNVII palsy, communicating hydrocephalus | chondroid | NA |
| Agunbiade et al. | clivus | unspecified resection | transnasal, with lateral cervical approach | NA | chondroid | NA |
| Asano et al. | clivus | unspecified resection | left posterior transpetrosal | NA | conventional | NA |
| Auger et al. | clivus | none | NA |  | NA | cytokeratin |
| Aydin et al. | clivus | unspecified resection | NA | NA | NA | NA |
| Boyette et al. | clivus | complete resection | transoral palate-splittingapproach | NA | NA | NA |
| Brooks et al. | clivus | none | NA | NA | NA | NA |
| Couldwell et al. | clivus, occipital condyle, left posterior fossa | subtotal resection | suboccipital craniectomy | NA | NA | cytokeratin, epithelial membrane antigen, S100 |
| Dahl et al. | clivus | biopsy only | NA | NA | chondroid | epithelial membrane antigen, S100, nu- clear brachyury expression, nuclear INI-1 expression |
| Figueiredo et al. | "pineal mass" | unspecified resection | supracerebellar infratentorial access | NA | NA | NA |
| Fischbein et al. | clivus | complete resection | right trans-sphenoethmoidal approach | NA | NA | NA |
| Fischbein et al. | clivus | subtotal resection | sublabial transsphenoidal | unilateral blindness | NA | NA |
| Fischbein et al. | clivus | subtotal resection | transoral | NA | NA | NA |
| Goes et al. | clivus | subtotal resection | NA | NA | NA | NA |
| Hines et al. | clivus | subtotal resection | transseptal | NA | NA | NA |
| Iloreta et al. | clivus to C5 | complete resection | left anterior cervical followed by posterior | NA | NA | NA |
| Jain et al. | skull base near foramen magnum to C1, C2 | none | NA | NA | NA | cytokeratin, vimentin, S-100 |
| KANEKO et al. | clivus | subtotal resection | transpetrosal, transtentorial | NA | NA | epithelial membrane antigen, vimentin, CK |
| Kearns et al. | edge of foramen magnum, clivus | complete resection | lateral transcondylar | transient quadriparesis | conventional | p-S6RP, p-mTOR, p-AKT and PTEN, brachyury |
| Kim et al. | clivus, right jugular tubercule, retropharyngeal space | complete resection | transoral, transpalatal | postoperative wound dehiscence, communicating hydrocephalus | NA | NA |
| Krishnamurthy et al. | sella, clivus | unspecified resection | transsphenoidal | NA | poorly differentiated | cytokeratin, CK 19, epithelial membrane antigen (EMA), vimentin, S‐100 |
| Loehn et al. | spheno-occipital | unspecified resection | NA | NA | NA | NA |
| Lopez et al. | clivus | subtotal resection | NA | NA | NA | NA |
| Lountzis et al. | clivus | none | NA | NA | NA | S-100, cytokeratin, epithelial membrane antigen |
| Maira et al. | clivus, sphenoid sinus, sella (multiple primaries) | complete resection | transsphenoidal | NA | NA | NA |
| Martin et al. | clivus | unspecified resection | transsphenoidal | NA | NA | NA |
| Nor et al. | sellar, supraseller | complete resection | transsphenoidal | NA | NA | Vimentin, keratin, epithelial membrane antigen, brachyury |
| Ogi et al. | clivus | subtotal resection | NA | NA | NA | NA |
| Plese et al. | clivus | subtotal resection | frontotemporal craniotomy | hypovolemic shock | NA | NA |
| Renard et al. | clivus | biopsy only | NA | NA | NA | cytokerattin AE1/AE3, epithelial membrane antigen, brachychury |
| Rutkowski et al. | clivus | unspecified resection | transoral | meningitis, CSF leak | NA | cytokeratin, epithelial membrane antigen |
| Schonegger et al. | clivus infiltrating sinus sphenoid | complete resection | NA | NA | chondroid | NA |
| Shakir et al. | foramen magnum | subtotal resection | left far lateral | left hypoglossal palsy and some increased difficulty in swallowing | conventional | S-100, cytokeratin, brachyury |
| Sibley et al. | spheno-occipital - vertebral | none | NA | NA | NA | cytokeratin, vimentin, epithelial membrane antigen |
| Uggowitzer et al. | clivus | subtotal resection | NA | NA | chondroid | NA |
| UHR et al. | sella turcica | none | NA | NA | NA | NA |
| van Lierop et al. | clivus | subtotal resection | transoral transpalatal | NA | NA | NA |
| Yasue et al. | clivus | biopsy only | NA | NA | poorly differentiated | NA |
| Zemmoura et al. | clivus | subtotal resection | sublabial transrhinoseptal | NA | NA | NA |
| Zener et al. | clivus | unspecified resection | NA | NA | NA | NA |
