# Supplementary material for: Metastatic skull base chordoma: A systematic review
Source: Laryngoscope Investig Otolaryngol. 2022 Sep 9;7(5):1280–91. doi: 10.1002/lio2.906 (PMC9575061; doi:10.1002/lio2.906)
Supplement: Supplementary file 4 — APPENDIX S4 Further information on skull base chordoma metastases [file LIO2-7-1280-s004.docx]

| **Appendix S4.** Further Information on Skull Base Chordoma Metastases | | | | | | |
| --- | --- | --- | --- | --- | --- | --- |
| Authors | Time from Primary to Metastasis  (Months) | Primary Metastases, All Metastases | Metastatic Imaging (Modality and Description) | Biopsy of Metastasis | Symptoms of Metastasis | Treatment of Metastasis |
| Agrawal et al. | NA | lungs, M1: lungs | M1: CXR. multiple cannonball lesions in both lungs | NA | NA | RT |
| Agunbiade et al. | concurrent | lungs, M1: lungs | M1: MRI/CT. multiple lung nodules | NA | NA | NA |
| Asano et al. | M1: 132 | bone, M1: T4, T8, T9, lumbar vertebra | M1: MRI. multiple mass lesions located in thoracic and lumbar vertebra | M1: yes | M1: sudden lower L extremity pain, para-paresis, bladder/bowel incontinence | laminectomy, RT |
| Auger et al. | concurrent | lungs, M1: lungs | M1: CXR/CT. bilateral lung densities | M1: yes | NA | NA |
| Aydin et al. | M1: 60 M2: 90 | bone, M1: T2, T3 M2: C1, C2 | M1: MRI/CT/PET. Intra-spinal canal mass T2, T3 level M2: MRI. diffuse signal abnormality within the C1 and C2 vertebral bodies | M1: yes M2: no | M1: weakness and numbness of lower extremities, back pain, paraparesia | M1: T3 total laminectomy M2: C1, C2 laminectomy |
| Boyette et al. | M1: 41 | soft tissue, M1: left retropharyngeal space | M1: MRI. ring enhancing area left retropharyngeal space | M1: yes | M2: swelling of left parotid area | M2: total resection |
| Brooks et al. | M1: 7 | lungs, M1: lungs | M1: autopsy | M1: yes | NA | NA |
| Couldwell et al. | M1: 28 | soft tissue, M1: subcutaneous nodules in back, abdomen | M1: CT. peritoneal masses | NA | NA | NA |
| Dahl et al. | concurrent | soft tissue, M1: chest wall | M1: NA | M1: yes | NA | NA |
| Figueiredo et al. | M1: 208 | soft tissue, M1: abdomen (mesogastrium) | M1: US. abdominal mass around VP catherer | yes | abdominal pain, constipation, distention | NA |
| Fischbein et al. | M1: 24 | soft tissue, M1: subcutaneous mass right bridge of nose | M1: mass along the right anterior nasal septum | yes | NA | M1: resection, RT |
| Fischbein et al. | M1: 49 | soft tissue, M1: anterior nasal mass | M1: MRI. mass involving the right bridge of the nose | yes | NA | M1: resection, RT |
| Fischbein et al. | M1: NA | bone, M1: anterior ethmoid | M1: MRI. large soft tissue mass anterior ethmoid | NA | NA | M1: resection |
| Goes et al. | M1: 36 M2: 48 | soft tissue, M1: subcutaneous medial right neck M2: C4, C5 | M1: NA M2: MRI. extravertebral 3 cm lobular process extending in the foramen of C4/C5 | M1: NA M2: yes | M1: NA M2: weakness right deltoid muscle | M1: resection M2: subtotal resection, RT |
| Hines et al. | M1: NA | soft tissue, M1: left nasal cavity | M1: MRI. 2.8 × 2.5-cm mass left nasal cavity | M1: yes | M1: NA | M1: non-operative, medical management |
| Iloreta et al. | M1: 73 | soft tissue, M1: anterior left neck incision site | M1: MRI. mass anterior to left sternocleidomastoid muscle | M1: yes | M1: NA | M1: resection with wide margins |
| Jain et al. | concurrent | soft tissue, M1: nasopharynx | M1: CT. 67 x 22mm mass superior nasopharynx | M1: yes | dysphagia, dysarthria, sore throat | M1: radiation |
| Kaneko et al. | M1: 9 M2: 9 M3: 9 M4: 9 M5: 9 M6: 9 M7: 9 | disseminated, M1: dura mater M2: skull bone M3: lungs M4: liver M5: sternum M6: left humerus M7: vertebrae | M1: autopsy M2: autopsy M3: autopsy M4: autopsy M5: autopsy M6: autopsy M7: autopsy | M1: yes M2: yes M3: yes M4: yes M5: yes M6: yes M7: yes | NA | NA |
| Kearns et al. | M1: concurrent M2: NA | lungs, M1: lungs M2: subcutaneous tissue, neck at incision site | M1: CT. 16 metastasis lung base M2: MRI. 2cm mass in soft tissue under surgical scar | M1: no M2: yes | M1: NA M2: sleep position and sleep difficulty, difficulty holding head upright (tumor grew to ∼35×20×14 cm) | M1: chemotherapy (ifosfamide, vincristine, actinomycin D, doxorubicin), |
| Kim et al. | M1: 51 | soft tissue, M1: heart | M1: PET-CT. multiple focal lesions. MRI. Large tumor in the right ventricular inferior wall and 2 lesions in the left ventricular lateral wall | yes | exertional dyspnea | M1: palliative chemotherapy (imatinib x2 weeks before dying) |
| Krishnamurthy et al. | M1: concurrent M2: 1 M3: 1 | bone, M1: left hemimandible M2: lungs M3: right mandible | M1: CT. lytic lesion in the left hemimandible with a large exophytic soft tissue component M2: NA M3: NA | NA | swelling of left lower face | M1: resection M2: supportive therapy |
| Loehn et al. | M1: 48 M2: 72 M3: 72 M4: 72 M5: 72 | soft tissue, M1: neck M2: mandible  M3: lungs M4: vertebral column M5: axial skeleton | M1: NA M2: MRI. 5-cm diameter right mandibular soft tissue mass M3: PET-CT M4: PET-CT M5: PET-CT | M1: yes M2: yes M3: no M4: no M5: no | M1: NA M2: NA M3-M5: pain in the neck and back and decreased vision in the right eye | M1: dissection, chemotherapy, RT M2: chemotherapy |
| Lopez et al. | M1: 24 M2: 25 | bone, M1: thoracic and lumbar spine M2: distal spinal cord and cauda equina nerve roots | M1: MRI M2: MRI. Numerous intradural drop metastases causing severe spinal canal stenosis with compression of the distal spinal cord and cauda equina nerve roots. | NA | M1: weakness, paresthesias, decreased sensation in the right lower extremity, right lower back/hop pain and paresthesias radiating down the leg M2: pain, progressive leg weakness, urinary retention, bowel incontinence | M1: radiation M2: radiation |
| Lountzis et al. | concurrent | disseminated, M1: skin M2: spinal cord M3: lungs M4: heart M5: liver M6: kidneys M7: brain parenchyma | M1: NA M2: NA M3: NA M4: NA M5: NA M6: NA M7: NA | M1: yes M2: no M3: no M4: no M5: no M6: no M7: no | M1: NA M2: NA M3: NA M4: NA M5: NA M6: NA M7: NA | M1: chemotherapy (cisplatin, doxorubicin, VP-16, vincristine, ifosphamide) M2: chemotherapy (same regimen) M3: chemotherapy (same regimen) M4: chemotherapy (same regimen) M5: chemotherapy (same regimen) M6: chemotherapy (same regimen) M7: chemotherapy (same regimen) |
| Maira et al. | NA | bone, M1: lumbar spine | NA | NA | NA | M1: resection |
| Martin et al. | M1: 144 | bone, M1: L4, L5, cauda equina | M1: MRI. lesion posterior to L5 vertebral body compressing the cauda equina and secondary stenosis from protrusion of the L4/5 disc | M1: yes | M1: lower limb weakness, right hip pain, post-void residual incontinence, ataxic gait | M1: resection, RT |
| Nor et al. | M1: 11 M2: 11 | bone, M1: foramen magnum M2: posterior epidural space L4, L5 | M1: MRI. small round, homogenously enhancing intradural extramedullary solid nodule M2: MRI. similar appearing nodular lesion in the posterior epidural space at the L4-5 level | NA | NA | M1: observation M2: observation |
| Ogi et al. | M1: 12 M2: NA M3: NA | soft tissue, M1: skin M2: lungs, lymph nodes M3: pelvic cavity | M1: MRI M2: NA M3: NA | M1: yes M2: no M3: no | M1: tender nodule medial left thigh M2: NA M3: NA | M1: resection M2: NA M3: NA |
| Plese et al. | NA | soft tissue, M1: subarachnoid space brain and spinal cord | M1: autopsy | NA | NA | NA |
| Renard et al. | M1: 18 | bone, M1: right costovertebral gutter | NA | M1: yes | NA | NA |
| Rutkowski et al. | M1: 55 M2: NA M3: NA M4: NA | lungs, M1: lungs M2: chest wall M3: calvaria M4: skin M5: chest wall | M1: PET/CT. isolated right lower lobe mass measuring 3.8 × 2.9 cm M2: NA M3: NA M4: NA M5: NA | M1: yes M2: no M3: no M4: yes M5: yes | M1: asymptomatic M2: NA M3: NA M4: skin breakdown and drainage M5: painful mass | M1: resection M2: chemotherapy (imatinib, sirolimus) M3: chemotherapy (imatinib, sirolimus) M4: resection, chemotherapy (nivolumab) M5: EZH2 inhibitor |
| Schonegger et al. | M1: 84 M2: 96 | lungs, M1: lungs M2: brain | M1: X-ray M2: MRI. Ring-shaped enhancing structure in left temporal lobe | NA | NA | M1: RT, chemotherapy (thalidomide, doxorubicin) M2: RT |
| Shakir et al. | concurrent | soft tissue, M1: breast | M1: X-ray. 2 cm mass left breast | M1: yes | NA | M1: mastectomy, RT |
| Sibley et al. | concurrent | lungs, M1: lungs | M1: pulmonary nodular infiltrates | M1: yes | M1: cough, tachypnea | M1: declined treatment |
| Uggowitzer et al. | M1: 42 M2: 78 M3: 78 | bone, M1: C4/5 intervertebral foramen M2: C5/6 intervertebral foramen M3: thoracic and lumbar spine | M1: MRI. 1.5 cm in diameter and transgressing the right C4/5 intervertebral foramen, with a dumbbell configuration M2: MRI. dumbbell metastasis in the left intervertebral foramen at C5/6.  M3: Small asymptomatic masses on thoracic and lumbar spine each measuring 8 mm | M1: yes M2: no M3: no | M1: asymptomatic mass M2: asymptomatic M3: asymptomatic | NA |
| UHR et al. | concurrent | lungs, M1: lungs | NA | M1: yes | M1: dyspnea, clubbing | NA |
| van Lierop et al. | M1: 24 | bone, M1: hard palate | M1: CT, MRI. 3 cm x 3 cm x 2.5 cm mass in midline of hard palate | M1: yes | M1: gradually enlarging asymptomatic palatal mass and nasal obstruction | M1: resection |
| Yasue et al. | concurrent | bone, M1: left upper arm M2: right iliac bone | M1: PET/CT. osteoblastic metastatic lesions  M2: PET/CT. osteoblastic metastatic lesions | NA | NA | M1: chemotherapy( vincristine, doxorubicin, cyclophosphamide, ifosfamide, carboplatin, etoposide) M2: chemotherapy (same regimen) |
| Zemmoura et al. | M1: 31 | bone, M1: maxilla | M1: CT: right upper maxillary bone defect between the 11th and 12th teeth | M1: yes | M1: painless loosening upper incisor | M1: resection |
| Zener et al. | NA | soft tissue, M1: left mid-sternocleidomastoid region | M1: MRI, CT. residual enhancement along the clival bone and left retropharyngeal area. | M1: yes | NA | M1: resection |
