# Supplementary material for: Metastatic skull base chordoma: A systematic review
Source: Laryngoscope Investig Otolaryngol. 2022 Sep 9;7(5):1280–91. doi: 10.1002/lio2.906 (PMC9575061; doi:10.1002/lio2.906)
Supplement: Supplementary file 5 — APPENDIX S5 Further information on skull base chordoma recurrences [file LIO2-7-1280-s002.docx]

| **Appendix S5**. Further Information on Skull Base Chordoma Recurrences | | | |
| --- | --- | --- | --- |
| Authors | Number of Local Recurrences | Time from Primary to Recurrence (Months) | Treatment of Recurrence |
| Agrawal et al. | 3 | NA | P1: subtotal resection P2: subtotal resection  P3: RT |
| Asano et al. | 2 | P1: 84 P2: 104 | P1: subtotal resection, GammaKnife P2: subtotal resection, RT |
| Boyette et al. | 3 | P1: 4 P2: 37 P3: NA | P1: total resection, RT P2: total resection P3: NA |
| Couldwell et al. | 2 | P1: 12 P2: 32 | P1: total resection P2: total resection |
| Figueiredo et al. | 1 | P1: 208 | none |
| Fischbein et al. | 1 | NA | GammaKnife |
| Fischbein et al. | 2 | P1: 12 P2: NA | P1: subtotal resection P2: resection, RT |
| Goes et al. | 1 | P1: 6 | P1: resection, RT |
| Hines et al. | 1 | P1: NA | P1: non-operative, medical management |
| Iloreta et al. | 1 | P1: 22 | P1: Resection, RT |
| Kaneko et al. | 1 | P1: 1 | P1: resection |
| Kearns et al. | 2 | P1: 18 P2: NA | P1: total resection P2: chemotherapy (Imatinib followed by etoposide) |
| Loehn et al. | 1 | P1: 48 | P1: chemotherapy, RT, resection |
| Lopez et al. | NA | NA | NA |
| Martin et al. | 1 | P1: 144 | NA |
| Ogi et al. | 1 | P1: 12 | NA |
| Schonegger et al. | 3 | P1: 12 P2: 36 P3: 48 | P1: resection P2: resection P3: resection, RT, etoposide, ifosfamide, doxorubicin, vincristine, isotretinoin, interferon-alpha |
| Uggowitzer et al. | 1 | P1: 3 | NA |
| Yasue et al. | 1 | P1: 15 | P1: resection, RT |
| Zemmoura et al. | 2 | NA | P1: resection P2: resection |
